# Recent Advancements for the Treatment of Hepatocellular Carcinoma

**DOI:** 10.3390/cancers15184444

**Published:** 2023-09-06

**Authors:** Kazuhiro Nouso

**Affiliations:** Department of Gastroenterology, Okayama City Hospital, 3-20-1, Kitanagase-omotemachi, Kita-ku, Okayama 700-8557, Japan; kazunouso@gmail.com

Hepatocellular carcinoma (HCC) is the third leading cause of cancer-related deaths worldwide and is currently one of the most common cancers [1]. Recently, dramatic changes have occurred in clinical practice regarding HCC. The most prominent change was in the treatment of HCC.

Surgical resection and ablation therapy are the two major approaches for treating early-stage HCC. Their adaptive criteria are now changing based on the results of several landmark studies such as the SURF study [2]. Both surgical resection and ablation therapies can now be used equally for the treatment of early-stage HCCs. Expanding the use of ablation for the treatment of metastatic lesions is another important topic [3].

The treatment of intermediate-stage HCC has also changed. Transcatheter arterial chemoembolization (TACE) has traditionally been used as the standard therapy for intermediate-stage HCC. Even with this established treatment, novel methods, such as balloon-occluded TACE, were developed, and higher effectiveness with reduced liver damage were achieved [4]. In contrast, owing to the high treatment efficacy of molecular targeted agents (MTAs) in intermediate-stage HCC, the latest HCC treatment guidelines recommend the use of MTAs in cases of diffuse, infiltrative, or extensive bilobar HCC [5,6].

Currently, many options are available for the treatment of advanced HCC. Sorafenib was the first MTA, and thereafter, multiple new drugs were developed [7]. These drugs present different landscapes in the treatment of HCC. Atezolizumab plus bevacizumab and durvalumab plus tremelimumab are the two major candidates for first-line treatment. While these drugs may not be effective for everyone, many of them are available for subsequent use. Moreover, multiple phase III studies are ongoing, and new concept therapies such as immune therapy following ablation therapy have emerged [7].

There were also significant recent advances in radiation therapy. Stereotactic body radiotherapy (SBRT) can be used to treat HCC effectively with minimal invasiveness [8]. Due to the aging of patients with HCC, the need for minimally invasive radiation therapy is increasing [9].

In this Special Issue, leading doctors in each field review the recent advancements in HCC treatment. The reports explain the latest status of these advancements so that they will serve as good guides for clinicians to properly treat HCC.
